# Potential Impact of the Involvement of Clinical Pharmacists in Antimicrobial Stewardship Programs on the Incidence of Antimicrobial-Related Adverse Events in Hospitalized Patients: A Multicenter Retrospective Study

**DOI:** 10.3390/antibiotics10070853

**Published:** 2021-07-14

**Authors:** Yewon Suh, Young-Mi Ah, Ha-Jin Chun, Su-Mi Lee, Hyung-sook Kim, Hyun-Jun Gu, A-Jeong Kim, Jee-Eun Chung, Yoonsook Cho, Young-Hee Lee, Shin-Yi Hwangbo, Jeongmee Kim, Eu-Suk Kim, Hong-Bin Kim, Eunsook Lee, Ju-Yeun Lee

**Affiliations:** 1 College of Pharmacy and Research Institute of Pharmaceutical Sciences, Seoul National University, Seoul 08826, Korea; yyse@snubh.org; 2 Department of Pharmacy, Seoul National University Bundang Hospital, Seongnam 13620, Korea; kehese2956@naver.com (H.-s.K.); escduck@snubh.org (E.L.); 3 College of Pharmacy, Yeungnam University, Gyeongsan 38541, Korea; ymah@ynu.ac.kr; 4 Department of Pharmacy, Ajou University Medical Center, Suwon 16499, Korea; hjchun@aumc.ac.kr (H.-J.C.); yh03@aumc.ac.kr (Y.-H.L.); 5 Department of Pharmacy, Samsung Medical Center, Seoul 06351, Korea; sumii.lee@samsung.com (S.-M.L.); jeongmee.kim@samsung.com (J.K.); 6 Department of Pharmacy, The Catholic University of Korea Seoul ST. Mary’s Hospital, Seoul 06591, Korea; guhj@cmcnu.or.kr (H.-J.G.); maricaren@cmcnu.or.kr (S.-Y.H.); 7 Department of Pharmacy, Seoul National University Hospital, Seoul 03080, Korea; anemone@snuh.org (A.-J.K.); joys99@snuh.org (Y.C.); 8 College of Pharmacy and Institute of Pharmaceutical Science and Technology, Hanyang University, Ansan 15588, Korea; jechung@hanyang.ac.kr; 9 Department of Internal Medicine, Seoul National University College of Medicine, Seoul National University Bundang Hospital, Seongnam 13620, Korea; eskim@snubh.org (E.-S.K.); hbkimmd@snubh.org (H.-B.K.)

**Keywords:** antimicrobial agents, diarrhea, nephrotoxicity, hepatotoxicity, thrombocytopenia, neutropenia, allergic reaction

## Abstract

Although specialized pharmacists have been suggested to be essential members of antimicrobial stewardship programs (ASPs), not all hospitals in Korea operate ASPs with pharmacists involved. We aimed to evaluate the association of involvement of clinical pharmacists as team members of multidisciplinary ASPs with the incidence of antimicrobial-related adverse drug events (ADEs). Five tertiary teaching hospitals participated in this retrospective cohort study. At each participating hospital, we randomly selected 1000 participants among patients who had received systemic antimicrobial agents for more than one day during the first quarter of 2017. We investigated five categories of antimicrobial-related ADEs: allergic reactions, hematologic toxicity, nephrotoxicity, hepatotoxicity, and antimicrobial-related diarrhea. Multivariate logistic regression analysis was used to evaluate the potential impact of pharmacist involvement in ASPs on the incidence of ADEs. A total of 1195 antimicrobial-related ADEs occurred in 618 (12.4%) of the 4995 patients included in the analysis. The overall rate of ADE occurrence was 17.4 per 1000 patient days. Hospitals operating ASPs with pharmacists showed significantly lower AE incidence proportions than other hospitals (8.9% vs. 14.7%; *p* < 0.001). Multidisciplinary ASPs that included clinical pharmacists reduced the risk of antimicrobial-related ADEs by 38% (adjusted odds ratio 0.62; 95% confidence interval 0.50–0.77). Our results suggest that the active involvement of clinical pharmacists in multidisciplinary ASPs may contribute to reduce the incidence of antimicrobial-related ADEs in hospitalized patients.

## 1. Introduction

Antimicrobial agents belong to the most frequently prescribed medications, and approximately 38–85% of hospitalized patients are prescribed at least one antimicrobial agent, with the highest rate of prescriptions in intensive care settings [1,2,3,4]. Although antimicrobial therapy is essential for treating or preventing infectious diseases, it is well known that overuse or misuse of antimicrobial agents causes antimicrobial resistance and antimicrobial-related adverse events [5,6,7,8].

Adverse drug events (ADEs) may result in hospitalization, prolonged hospital stay, disability, or even death [9,10], and antimicrobial use is inevitably associated with potentially adverse effects such as gastrointestinal toxicity including *Clostridioides difficile* infection (CDI), nephrotoxicity, hepatotoxicity, and neurotoxicity, some of which are preventable [11]. Few data are available regarding the estimated incidence of antimicrobial-related ADEs among hospitalized patients; however, a previous single-center study reported that approximately 20% of hospitalized patients experienced antimicrobial-related ADEs [12].

The World Health Organization (WHO) proposed an antimicrobial stewardship program (ASP) which comprises interventions designed to promote the optimal use of antimicrobial agents as a global action plan [13]. Diverse governmental agencies and the US Centers for Disease Control and Prevention, the European Centre for Disease Prevention and Control, many EU countries, and Australia recommend implementing ASPs in all hospitals [14,15]. The main objective of ASPs is to address the growing problem of antimicrobial resistance; however, such programs may also help reduce the incidence of adverse events associated with antimicrobial use by reducing inappropriate antimicrobial use. The incidence of CDI has been reported to decrease as a result of ASP intervention [16]. However, few studies have comprehensively evaluated the effect of ASPs on the reduction of antimicrobial-related ADEs in general.

Most guidelines suggest that clinical pharmacists are essential members of ASPs [14,15]; however, in Korea, ASPs of numerous hospitals did not include clinical pharmacists, even though more than 90% of the hospitals in Korea have implemented ASPs, according to a previous survey [17]. Only a few hospitals execute ASPs with active involvement of pharmacists; therefore, we aimed to evaluate the potential impact of involvement of clinical pharmacists as team members of multidisciplinary ASPs on the incidence of antimicrobial-related ADEs.

## 2. Results

### 2.1. Population Characteristics

In total, 4995 patients were included in the analysis after excluding 5 patients due to incompleteness of laboratory data. Demographic data of the patients are presented in Table 1. The median age was 57 years (IQR 37–69 years). Pediatric patients (<18 years), older adults (≥65 years), and females accounted for 13.9%, 32.5%, and 47.3% of the study population, respectively. The median length of hospital stay was nine days (IQR 5–16 days). Cancer patients accounted for 36.6%, and 8.1% and 9.2% of the patients had kidney and liver diseases, respectively. Approximately 58% of the patients were prescribed monotherapy, and 11% of the patients were prescribed more than three antimicrobials. The median duration of antimicrobial administration was four days (IQR 2.5–7 days), with approximately 73% receiving antimicrobials for less than seven days. Hospitals operating ASPs with pharmacists had a significantly shorter length of hospital stay, fewer antimicrobials, and fewer days of antimicrobial therapy (*p* < 0.001) (Table 1). 

The most frequently prescribed antimicrobial class was that of third-generation cephalosporins (1730 patients; 34.6%), followed by first-generation cephalosporins (1123 patients; 22.5%), second-generation cephalosporins (1081 patients; 21.6%), and anti-pseudomonal penicillin with beta-lactamase inhibitor (750 patients; 15.0%).

### 2.2. Incidence of Antimicrobial Associated ADEs

A total of 1195 antimicrobial-related ADEs occurred in 618 patients. The incidence proportion of overall ADEs was 12.4%, ranging from 8.6% to 16.2%. The overall rate of ADE incidence was 17.4/1000 treatment days.

The ADEs with the highest incidence proportions were antimicrobial-related diarrhea (4.7%), thrombocytopenia (3.0%), nephrotoxicity (2.8%), hepatotoxicity (2.8%), and neutropenia (1.6%). Antimicrobial-related diarrhea that required pharmacological treatment occurred in 234 patients (4.7%), including patients diagnosed with CDI (113 patients; 2.3%). There were 68 cases of spontaneous allergic reactions reported in 52 patients (Table 2). 

The antimicrobials most frequently involved in ADEs among those used by more than 10 patients were tigecycline (incidence proportion 31.3%), voriconazole (30.4%), colistin (26.8%), azithromycin (21.1%), meropenem (20.9%), ganciclovir (20.6%), teicoplanin (20.3%), cefepime (20.1%), and linezolid (20.0%).

A total of 283 patients (5.7%) experienced moderate to severe ADEs, which accounted for 45.8% of the total patients who experienced ADEs. More than half of the patients with hepatotoxicity showed a severe grade of the affliction, while 71.6% of diarrhea cases were only mild. 

### 2.3. Potential Impact of Pharmacist Involvement on the Incidence of Antimicrobial-Related ADEs

Hospitals operating ASPs with pharmacists had a significantly lower ADE incidence proportion than other hospitals (8.9% vs. 14.7%; *p* < 0.001). The rates of ADE occurrence per 1000 treatment days were 15.1 and 18.3 in hospitals with ASPs with and without pharmacists, respectively (*p* = 0.003). Multivariate logistic analysis showed that multidisciplinary ASPs including clinical pharmacists reduced the risk of antimicrobial-related ADEs by 38% (adjusted odds ratio 0.62; 95% confidence interval 0.50–0.77) after adjusting for known factors associated with antimicrobial ADEs (age, sex, length of hospital stay, stay in the intensive care unit, cancer, kidney disease, hepatic disease, diabetes mellitus, and number of antimicrobial agents) (Table 3).

## 3. Discussion

We found that approximately 12.4% of patients using antimicrobial agents experienced adverse events, and approximately 17.4 adverse events were confirmed per 1000 antimicrobial prescriptions. We also demonstrated that involvement of a clinical pharmacist in an ASP reduced the risk of antimicrobial-related ADEs.

The ADE incidence proportion in this study (12.4%) was somewhat lower than that reported in a previous study (20%), which retrospectively estimated adverse events associated with antimicrobial use in 1488 adults at a university hospital in Maryland, USA [12]. This may be explained by the difference in the study population and the difference in the definition and method of identifying ADEs. We included pediatric patients and targeted specific major adverse events that may be objectively identified using the existing data. Therefore, adverse events such as cardiac complications, neurological afflictions, muscle pain, nausea, and vomiting were not considered in the present study. 

Few studies have reported the general incidence of ADEs associated with antimicrobials in hospitalized patients. A retrospective cohort study on antimicrobial-related ADEs in a single hospital in Korea showed the highest incidence of allergic reactions [18], which is presumably due to the high reporting rate of easily recognized ADEs such as skin reactions. However, the authors were unable to estimate the incidence rate in all patients using antimicrobial agents. 

Our results provide an estimate of the incidence of antimicrobial-related ADEs in hospitalized patients using antimicrobial agents from a multicenter study, and among antimicrobial-related ADEs, antimicrobial-related diarrhea showed the highest incidence (in 4.7% of the patients), followed by thrombocytopenia, nephrotoxicity, and hepatotoxicity. Allergic reactions identified by spontaneous reports occurred in 1.0% of the study patients, indicating that the prevalence type of ADEs depends on the method of study, i.e., spontaneous reporting or retrospective chart review.

The number of antimicrobials and treatment days and the length of stay were lower in hospitals operating ASP with clinical pharmacists. This might be associated with ASP activities and differences in patient characteristics. Multivariate logistic regression also showed that the number of antimicrobial agents and age, long hospital stay, and comorbidity are associated with antimicrobial-related ADEs. We were unable to assess causal relationships; however, longer duration of hospitalization may result from ADEs associated with antimicrobial agents. The use of more than three antimicrobial agents increased ADE risk 7.4-fold. As a preventive measure, it is necessary to assess the possibility of discontinuing antimicrobial therapy as much as possible to reduce excessive use of antimicrobial agents.

Some studies reported that ASPs are effective for achieving appropriate use of antimicrobial agents [19,20,21,22]. To the best of our knowledge, this study is the first multicenter study to investigate the potential impact of pharmacist-involved multidisciplinary ASPs on antimicrobial-related ADEs. Participation of pharmacists reduced antimicrobial-related ADEs by 38%, which was in line with a previously observed effect of pharmacist intervention to reduce ADEs [23]. In addition, our findings support the recommendation for ASP measures to prevent ADEs associated with antimicrobial agents and for involving pharmacists specialized in infections as essential members of ASPs [11,14,15].

Our study has, however, a few limitations. First, due to the retrospective nature of this research, only predefined specific ADEs associated with antimicrobial agents were evaluated. If the patient did not revisit the study hospital, the incidence of CDI may also be underestimated despite data collection after discharge. Therefore, the overall incidence of ADE may be underestimated. However, applying the same ADE screening criteria on objectively collected data allowed us to determine the potential impact of pharmacist involvement and thereby reduced bias introduced by differences between hospital records. Second, there were more patients with comorbidities in hospitals without pharmacist involvement, which made patients vulnerable to antimicrobial-related ADEs. Even though we did the multivariable analysis adjusting major diseases to minimize the effect of known measurable confounders, the effect of unconsidered comorbid conditions on the incidence of antimicrobial-related ADEs could not be excluded. In addition, other factors for hospital-level could affect the incidence of ADE; however, we could not consider them. Third, since ADE assessed as ‘possible’ or stronger causality was considered to indicate an ADE, the potential influence of confounding variables such as comorbid disease, other concomitant medications, and drug–drug interactions could not be completely excluded. Finally, we cannot generalize our findings on a national or global scale as our results originate from only five tertiary teaching hospitals.

We showed that approximately 17% of hospitalized patients using antimicrobials experienced ADEs, and involvement of clinical pharmacists in multidisciplinary ASPs may contribute to reduce the incidence of antimicrobial-related adverse events in hospitalized patients.

## 4. Materials and Methods

### 4.1. Study Design and Setting

We conducted this retrospective cohort study at five tertiary teaching hospitals in Korea. The average number of inpatient beds was 1620, ranging between 1262 and 2129. The number of pharmacists ranged from 56 to 149 and pharmacists in all hospitals performed therapeutic drug monitoring (TDM) of antimicrobials. The ASP of all included hospitals consisted of infectious disease specialists, nurses, and laboratory microbiology specialists. We classified hospitals by whether they operated ASPs with or without the involvement of clinical pharmacists specialized in infectious diseases. Two hospitals executed an ASP in which at least one full-time clinical pharmacist dedicated to the ASP was actively involved. Clinical pharmacists dedicated to the ASP intervened in antimicrobial prescriptions and monitored antimicrobial-related ADEs in addition to TDM. 

### 4.2. Study Population and Data Collection

Among patients who were hospitalized between January and March 2017 and who were administered systemic antimicrobial agents for at least 24 h, one thousand patients were randomly selected at each of the five participating hospitals. When patients used systemic antimicrobial agents in at least two treatment periods during hospitalization, only the first treatment episode was included. The first date of systemic antimicrobial therapy was defined as the index date. Systemic antimicrobials included antibacterials (J01), antimycotics (J02), and antivirals for systematic use (J05AA, J05AB, J05AC, J05AD, and J05AH), according to the WHO anatomical therapeutic chemical classification. We excluded antituberculosis drugs and antiviral agents for hepatitis and HIV treatment. Demographic data, diagnosis, intensive care unit stay, length of hospital stay, antimicrobial regimen, days of antimicrobial therapy, medication use within three months after the index date, laboratory data, and records of spontaneous ADEs were retrieved from the electronic medical record system of each hospital. 

### 4.3. Definition of Antimicrobial-Related Adverse Events

Considering the retrospective nature of this study, we only included five categories of antimicrobial-related ADEs which can be detected objectively using the available data in order to minimize biases that may arise from deviations in the records of physicians, including allergic reactions, hematologic toxicity, nephrotoxicity, hepatotoxicity, and antimicrobial-related diarrhea including CDI. The operational definition of these adverse events was determined based on previous studies (Table 4) [24,25,26,27,28,29,30,31]. Using the modified method of Tamma et al. [12], we identified ADEs until the discharge date or 30 days after the start of antimicrobial agent treatment, whichever occurred first; however, for identification of CDI, we reviewed laboratory data including outpatient visits and readmission data after discharge up to 90 days after the start of antimicrobial treatment if patients returned to study hospital.

A two-step approach was adopted for identifying antimicrobial-related ADEs: first, potential ADE cases were screened using a computerized program with specific criteria such as laboratory results and the use of specific agents for management of ADEs; second, cases that met the criteria and those with voluntary reports of adverse events were reviewed by pharmacists, based on full individual electronic medical rec-ords. The pharmacists assessed causality according to the criteria of the WHO–Uppsala Monitoring Center, and causality considered ‘possible’ or stronger was considered to indicate an ADE. Severity was assessed using criteria suggested in previous studies (Table 5) [32,33,34].

### 4.4. Statistical Analyses

The proportion of incidences of antimicrobial-related ADEs was calculated as the numerator of patients who experienced ADEs divided by the number of patients who received antimicrobials. The incidence rate per 1000 treatment days was calculated using the numerator of the total number of ADE cases. Treatment days was defined as the sum of days of antimicrobial treatment of the included patients. If the patient received two or more antimicrobials on one day, the treatment days was counted as one day. The overall incidence and incidence rates were compared between ASPs with and without a pharmacist. Multivariate logistic regression analysis was used to evaluate the potential impact of pharmacist involvement in ASPs on the incidence of ADEs by adjusting for known factors associated with antimicrobial ADEs (age, sex, length of hospital stay, stay in the intensive care unit, cancer, kidney disease, hepatic disease, diabetes mellitus, and number of antimicrobial agents). Categorical variables were expressed as frequencies and percentages, and continuous variables were expressed as medians and interquartile range (IQR). The difference between ASPs with and without pharmacist was tested using a chi-square test. Statistical analyses were performed using SAS (version 9.4; SAS Institute, Inc., Cary, NC, USA).

## Figures and Tables

**Table 1:** Baseline characteristics of the patients.

Characteristics	Total (*n* = 4995) *n* (%)	ASP with Pharmacist (*n* = 2000) *n* (%)	ASP without Pharmacist (*n* = 2995) *n* (%)	*p*-Value
**Age**				0.254
<18 years	696 (13.9)	290 (14.5)	406 (13.6)	
18–64 years	2677 (53.6)	1086 (54.3)	1591 (53.1)	
≥65 years	1622 (32.5)	624 (31.2)	998 (33.3)	
**Sex**, female	2362 (47.3)	980 (49.0)	1382 (46.1)	0.048
**Stay in the Intensive Care Unit**	402 (8.1)	166 (8.3)	236 (7.9)	0.593
**Length of Hospital Stay**				<0.001
<15 days	3448 (69.0)	1608 (80.4)	1840 (61.4)	
15–21 days	897 (18.0)	228 (11.4)	669 (22.3)	
≥22 days	650 (13.0)	164 (8.2)	486 (16.2)	
**Diagnosis**				
Cancer	1829 (36.6)	467 (23.4)	1362 (45.5)	<0.001
Kidney disease	403 (8.1)	89 (4.5)	314 (10.5)	<0.001
Hepatic disease	457 (9.2)	106 (5.3)	351 (11.7)	<0.001
Diabetes mellitus	678 (13.6)	141 (7.1)	537 (17.9)	<0.001
**Number of Antimicrobials**				<0.001
1	2881 (57.7)	1086 (54.3)	1795 (59.9)	
2–3	1568 (31.4)	726 (36.3)	842 (28.1)	
≥4	575 (11.5)	188 (9.4)	387 (12.9)	
**Days of Antimicrobial Therapy**				<0.001
<7 days	3624 (72.6)	1628 (81.4)	1996 (66.6)	
7–13 days	1054 (21.1)	308 (15.4)	746 (24.9)	
14–20 days	211 (4.2)	41 (2.1)	170 (5.7)	
≥21 days	106 (2.1)	23 (1.2)	83 (2.8)	

**Table 2:** Incidence rate by type of adverse events and type of antimicrobial stewardship program.

	Total ^a^	ASP with Pharmacist	ASP without Pharmacist	*p*-Value
**Overall**				
Number of patients investigated	4995	2000	2995	
Patient prescribed antibiotics-days (patient-days)	68,803	20,363	48,440	
Onset time, days from index date, median (IQR)	4 (2–7)	3 (2–6)	4 (2–7)	
Incidence proportion	12.37	8.85	14.72	<0.001
Incidence rate per 1000 treatment days	17.37	15.08	18.33	0.003
**Diarrhea**				
Number of patients investigated	4995	2000	2995	
Patient prescribed antibiotics-days (patient-days)	68,803	20,363	48,440	
Onset time (diarrhea), days from index date, median (IQR)	4 (2–8)	4 (1–13.5)	4 (3–8)	
Onset time (CDI), days from index date, median (IQR)	10 (4–23)	6 (4–15)	12.5 (6–39.5)	
Incidence proportion (diarrhea)	4.68	2.85	5.91	<0.001
Incidence proportion (CDI)	2.26	2.05	2.40	0.410
Incidence rate per 1000 treatment days (diarrhea)	6.60	5.25	7.16	0.005
Incidence rate per 1000 treatment days (CDI)	3.53	4.22	3.24	0.047
**Nephrotoxicity**				
Number of patients investigated	4872	1966	2906	
Patient prescribed antibiotics-days (patient-days)	66,717	20,001	46,716	
Onset time, days from index date, median (IQR)	4 (2–6)	3 (2–6)	4 (2–6.5)	
Incidence proportion	2.81	2.29	3.17	0.069
Incidence rate per 1000 treatment days	3.25	2.95	3.38	0.369
**Hepatotoxicity**				
Number of patients investigated	4905	1983	2922	
Patient prescribed antibiotics-days (patient-days)	66,937	20,382	46,555	
Onset time, days from index date, median (IQR)	5 (3–11)	4 (2–11.5)	5 (3–11)	
Incidence proportion	2.75	1.41	3.66	<0.001
Incidence rate per 1000 treatment days	3.02	1.91	3.50	0.001
**Thrombocytopenia**				
Number of patients	4270	1949	2321	
Patient prescribed antibiotics-days (patient-days)	42,978	19,099	23,879	
Onset time, days from index date, median (IQR)	3 (2–6)	4 (2–6)	3 (2–5)	
Incidence proportion	2.95	1.95	3.79	<0.001
Incidence rate per 1000 treatment days	3.63	2.15	4.82	<0.001
**Neutropenia**				
Number of patients	4321	1924	2397	
Patient prescribed antibiotics-days (patient-days)	45,633	18,870	26,763	
Onset time, days from index date, median (IQR)	4 (3–6)	4 (2–6)	5 (3–9)	
Incidence proportion	1.60	1.35	1.79	0.249
Incidence rate per 1000 treatment days	2.15	1.64	2.50	0.051
**Allergic Reaction**				
Number of patients investigated	4995	2000	2995	
Patient prescribed antibiotics-days (patient-days)	68,803	20,363	48,440	
Onset time, days from index date, median (IQR)	1 (0–3.5)	1 (0–9)	1 (0–3)	
Incidence proportion	1.04	0.95	1.10	0.604
Incidence rate per 1000 treatment days	0.99	1.47	0.78	0.009

ASP, antimicrobial stewardship program; CDI, *Clostridioides difficile* infection. ^a^ The number of patients investigated was different for each ADE category because of exclusion criteria of each ADE category.

**Table 3:** Multivariate logistic analysis of factors associated with antimicrobial-related adverse drug events.

Characteristics	Adjusted Odds Ratio (95% CI)
**Age**	
<18 years	1
18–64 years	1.67 (1.19–2.36)
≥65 years	2.08 (1.47–2.96)
**Sex**	
Male	1
Female	0.97 (0.81–1.17)
**Length of Hospital Stay**	1.01 (1.01–1.01)
**Stay in the Intensive Care Unit**	
No	1
Yes	2.41 (1.85–3.16)
**Number of Antimicrobial Agents**	
1	1
2–3	2.92 (2.35–3.64)
≥4	7.36 (5.66–9.58)
**Cancer**	
No	1
Yes	1.24 (1.02–1.51)
**Kidney Disease**	
No	1
Yes	1.40 (1.05–1.87)
**Hepatic Disease**	
No	1
Yes	1.37 (1.04–1.81)
**Diabetes Mellitus**	
No	1
Yes	1.13 (0.89–1.45)
**Antimicrobial Stewardship Program**	
No	1
Yes	0.62 (0.50–0.77)

**Table 4:** Screening criteria of adverse events associated with antimicrobials.

Type of Adverse Events	Criteria of Adverse Events
Allergic reaction	Allergic reactions reported spontaneously during the study period
Hematologic toxicity	Neutropenia: ANC < 1500, Thrombocytopenia: Platelet < 100 × 103/㎕ Exclusion criteria Patients on anti-neoplastic therapy Neutropenia: baseline ANC < 1500 or WBC < 3000 cells/㎕ Thrombocytopenia: baseline platelet < 100 × 10^3^/㎕
Nephrotoxicity	Serum creatinine increased by 0.3 mg/dL or more after starting antimicrobials or by 1.5 times or more after starting antimicrobials Exclusion criteria Estimated glomerular filtration rate < 15 mL/min or dialysis
Hepatotoxicity	Baseline is within the normal range (1) ALT > upper limit of normal range (ULN) × 5 or (2) ALP > ULN × 2 (3) TB > ULN × 2 & ALT > ULN × 3 If baseline ALT is elevated (1) ALT > baseline ALT × 3 (2) ALP > baseline ALP × 2 LFT is more than 2 times higher than the upper limit of normal range, and when stopping antimicrobials at the physician’s judgment Exclusion criteria ALT elevation begins after antimicrobials are stopped Biliary tract stent treatment and liver transplantation within 3 days Diagnosed with viral hepatitis with abnormal laboratory test Baseline ALT is more than 5 times higher than the upper limit of the normal range
Antimicrobial associated diarrhea	Use of antidiarrhea drug (smectite, loperamide) and *C. difficile* toxin test negative or no test
*C. difficile* infection (CDI)	Occurs within 90 days of starting antimicrobials Positive result of *C. difficile* toxin test (ELISA or stool toxin) Oral metronidazole or oral vancomycin administration
	Exclusion criteria *C. difficile* toxin positive 60 days after the end of antimicrobials

ANC, absolute neutrophil count; WBC, white blood cell; ALT, alanine aminotransferase; ALP, alkaline phosphatase; TB, total bilirubin; LFT, liver function test.

**Table 5:** Criteria for evaluating the severity of toxicity according to the type of adverse events.

Type of Adverse Events	Severity	Definition
**Hematologic Toxicity**		
Neutropenia	mild	ANC 1000–1500
	moderate	ANC 500–999
	severe	ANC < 500
Thrombocytopenia	mild	Platelet 50–100 × 10^3^/㎕
	moderate	Platelet 30–50 × 10^3^/㎕
	severe	Platelet < 30 × 10^3^/㎕
Nephrotoxicity	mild	Less than twice the baseline
	moderate	2–3 times increase of baseline
	severe	More than 3 times the baseline
Hepatotoxicity	mild	ALT ≤ upper limit of normal range (ULN) × 3 or baseline ALT × 1.5–3
		ALP ≤ ULN × 2.5 or baseline ALP × 2.0–2.5
		TB ≤ ULN × 1.5 or baseline TB × 1.0–1.5
	moderate	ALT ≤ ULN × 3.0–5.0 or baseline ALT × 3–5
		ALP ≤ ULN × 2.5–5.0 or baseline ALP × 2.5–5.0
		TB ≤ ULN × 1.5–3.0 or baseline TB × 1.5–3.0
	severe	ALT > ULN × 5.0 or baseline ALT × 5.0
		ALP > ULN × 5.0 or baseline ALP × 5.0
		TB > ULN × 3.0 or baseline TB × 3.0
**Diarrhea**		
Antimicrobial associated diarrhea	mild	Duration of treatment: less than 4 days
	moderate	Duration of treatment: 4 ~ 7 days
	severe	Duration of treatment: more than 7 days
*C. difficile* infection (CDI) ^a^	mild	Treatment for less than 10 days with metronidazole alone
	moderate	Vancomycin oral treatment for less than 10 days or vancomycin oral treatment for less than 10 days after metronidazole treatment is started.
	severe	Vancomycin oral combined with metronidazole injection treatment or vancomycin enema

ANC, absolute neutrophil count; ALT, alanine aminotransferase; ALP, alkaline phosphatase; TB, total bilirubin. ^a^ The severity criteria of CDI was based on the 2010 version due to study period (2017).

## Data Availability

The data that support the findings of this study are available on request from the corresponding author.
